# Metachronous bladder metastases of a type 2 papillary renal cell carcinoma: a case report and review of the literature

**DOI:** 10.1186/s12957-016-0986-2

**Published:** 2016-08-22

**Authors:** D. G. V. D. Seneth Gajasinghe, Ishra Nazeer, Hansika P. Maddumage, Chrysantha Perera, Anuruddha M. Abeygunasekera

**Affiliations:** 1 Department of Urology, Colombo South Teaching Hospital, Dehiwala, Sri Lanka; 2 Department of Oncological Surgery, Karapitiya Teaching Hospital, Galle, Sri Lanka; 3 Department of Pathology, Colombo South Teaching Hospital, Dehiwala, Sri Lanka

**Keywords:** Papillary renal cell carcinoma, Bladder metastases, Kidney cancer

## Abstract

**Background:**

Renal cell carcinoma developing metastases in the bladder is rare. Bladder metastasis due to a papillary type of renal cell carcinoma is rarer. Such metastases could be synchronous or metachronous.

**Case presentation:**

Here we present a 55-year-old female patient with haematuria who underwent left nephro-ureterectomy for a suspected urothelial tumour. Histopathology revealed it to be a type 2 papillary renal cell carcinoma. Eighteen months later, she developed metachronous bladder metastasis of the papillary renal cell carcinoma which was treated with total cystectomy. Currently, she is on interferon therapy.

**Conclusions:**

These bladder metastases from renal cell carcinoma could be due to drop metastases, lymphatic spread or haematogenous spread. The exact mechanism in a given case appears to be unpredictable.

## Background

Renal cell carcinoma (RCC), the commonest renal malignancy in adult is known to have distant metastasis in about 20–30 % of patients at the time of diagnosis [1]. Another 20 % will develop metastasis during the follow-up [1]. Even early stage RCC confined to the kidney has the potential to develop metastases after surgery with curative intent. Common sites of metastases include the lung (50–60 %), lymph node (40–60 %), liver (30–40 %), and bone (30–40 %) [2]. Rare sites include the parotid gland, eye and urinary bladder [3]. About 65 cases have been published in the literature with bladder metastases of RCC [4]. Clear cell renal carcinoma is the commonest (92 %) histological type [4]. Only two cases of papillary type of renal cell carcinoma have been reported among those 65 cases. Here we describe a case of papillary type 2 carcinoma of the kidney developing metachronous metastases in the urinary bladder.

## Case presentation

A 55-year-old mother of two children developed haematuria, and the contrast enhanced computerised tomography of the urinary tract (CT KUB) showed a solid mass in the left kidney close to its hilum suggestive of a urothelial carcinoma of the renal pelvis. The mass was heterogenous with contrast enhancement and was 5.6 cm × 4.5 cm in dimensions. There was no vascular involvement or lymphadenopathy. The right kidney, ureters and bladder were normal. Cystoscopy did not show any mucosal lesions. She was a non-smoker and was having well-controlled hypertension. She had open left nephro-ureterctomy through loin and left lower abdominal incisions. The histology showed a type 2 papillary renal cell carcinoma—stage pT_1b_. Fuhrman grade was 3. She was followed up with no adjuvant therapy.

**Fig. 1:**
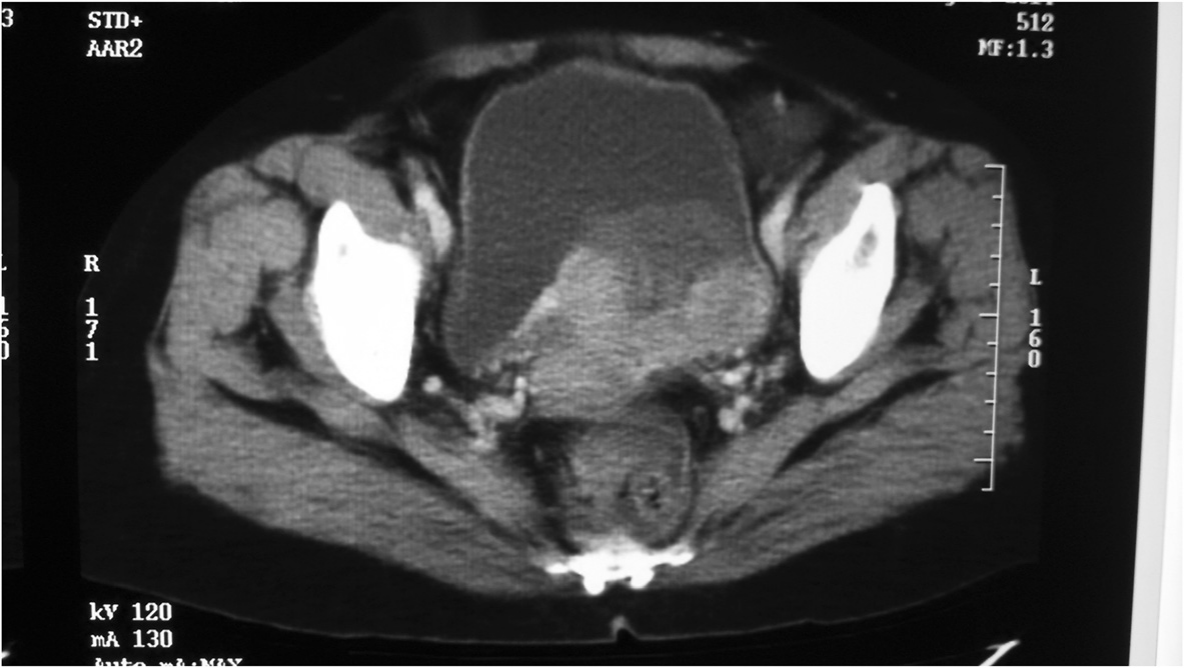
CT scan showing a large irregular mucosal lesion in the left base of the bladder

**Fig. 2:**
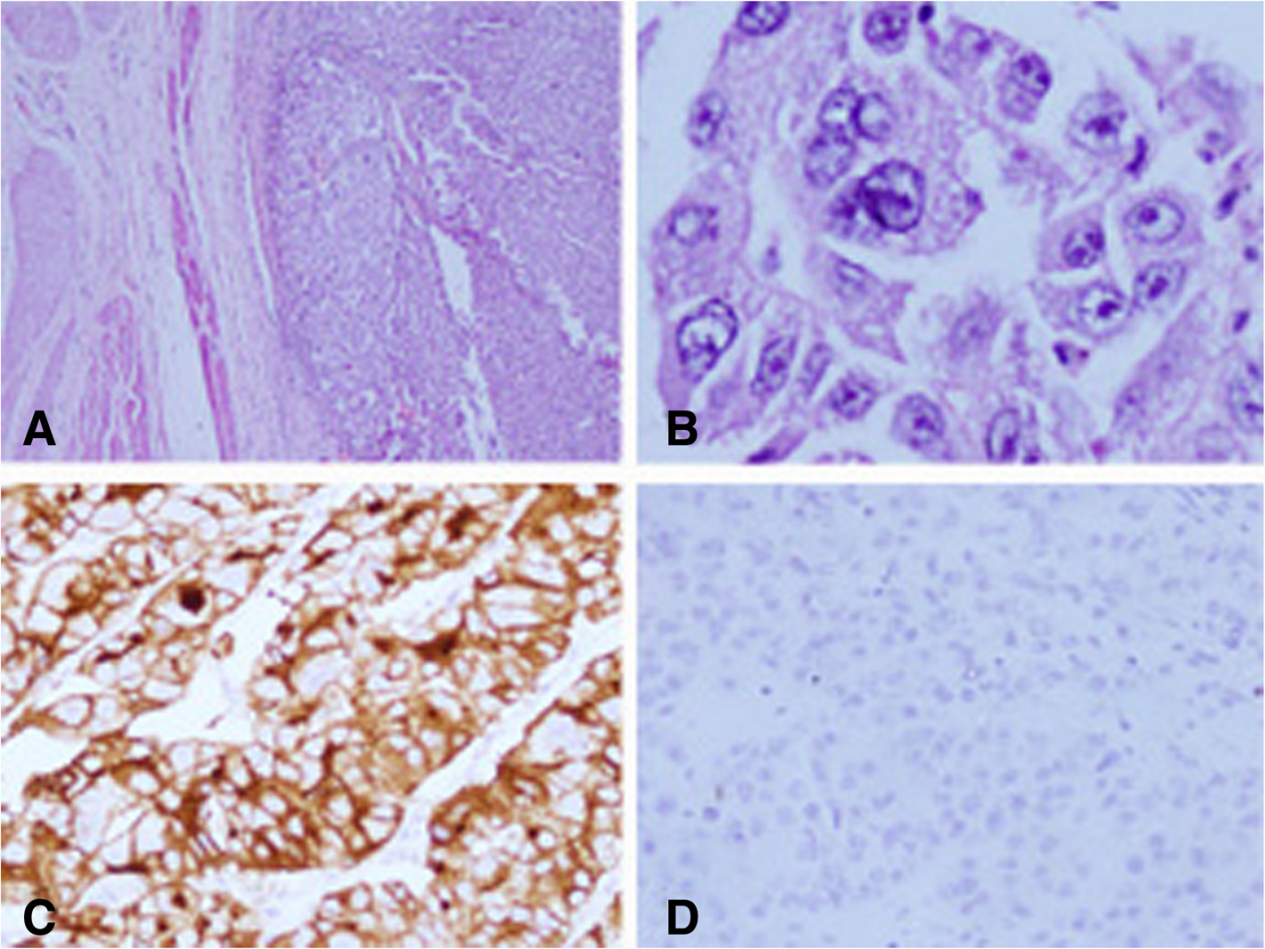
H&E staining and immunohistochemistry of the bladder lesion. **a** Papillary renal cell carcinoma involving the bladder wall (H&E stain, ×100). **b** Papillary renal cell carcinoma involving the bladder wall (H&E stain, ×200). **c** Immunohistochemistry showing strong membrane positivity for CD10 (×200). **d** immunohistochemistry showing negativity for CK20 (×200)

Eighteen months later, her routine follow-up US scan of KUB showed a mucosal lesion in the left base of the bladder which was confirmed by a CT scan (Fig. 1). It was confined to the bladder wall. She underwent transurethral resection of the bladder lesion which showed metastatic deposits of a high-grade papillary renal cell carcinoma (Figs. 2a and 2b). Immunohistochemistry showed strong membrane positivity for CD10 (Fig. 2c) while CK7 and CK20 were negative (Fig. 2d). Despite transurethral resection, the tumour was growing rapidly and she underwent total cystectomy and neobladder formation. Since then, she has interferon alpha injections thrice a week. Ten months after cystectomy, she developed a lung metastasis. She continues to have interferon and is well with an Eastern Cooperative Oncology Group (ECOG) performance status of 1, 6 months after the diagnosis of the lung metastasis.

### Discussion

Metastases to the urinary bladder occur in less than 1 % of solid tumours [5]. Renal cell carcinoma developing metastases in the bladder is extremely rare with an incidence of 0.3 % [6]. Clear cell carcinoma is the commonest histological variety of renal cell carcinoma that leads to bladder metastases [4]. Papillary carcinoma is less commonly involved, followed by collecting duct carcinoma and chromophobe cell carcinoma [4]. Hoffman reported the first case of renal cell carcinoma developing metastases in the bladder in 1907 [5]. Bladder metastases can be synchronous or metachronous. Metachronous metastases are commoner (77 %) [4]. The median time for metachronous bladder metastases following the diagnosis of renal cell carcinoma is 33 months [4]. The overall prognosis is poor in these patients with most patients dead within 1 year of diagnosing the bladder metastases.

Generally, papillary renal cell carcinoma has a less potential for metastases than clear cell carcinoma and has a better prognosis [7]. However, when metastases develop, papillary renal cell carcinoma behaves more aggressively than patients with a metastatic clear cell type of renal cell carcinoma [8]. The exact reason for this unusual behaviour is unknown.

The underlying mechanism of metastatic spread is debatable with several possible modes described [9]. One is “drop metastases” due to malignant cells moving along the urinary tract and getting implanted on the bladder epithelium [10]. This is supported by reports where the RCC deposit was free floating without attachment to the urothelium in histological examination and by the presence of cancer cells in urine of patients with RCC [11]. In others, bladder lesions are found along with metastatic deposits in other distant organs as well, suggestive of a hematogenous spread. However, another subgroup develops muscle-invasive or deep metastatic lesions in the bladder especially located near the ureteric orifice suggestive of spread along the lymphatics as emboli [12]. In these patients, retrograde venous embolisation of tumour cells is also a possibility [6]. In our patient, as the dissection of the kidney and ureter were done while the ureter was left patent and the fact that her primary lesion was close to the renal pelvis with haematuria as a symptom, drop metastases would have been more likely [13]. However, the lesion was deep in the bladder wall near the ureteric orifice which makes the peri-ureteric spread or hematogenous spread also possible. The latter mechanism is more plausible as she had a poorer prognosis with development of lung metastases within 10 months of surgery to remove bladder metastases. Those with drop metastases have a much better prognosis [14]. More than one mechanism of metastatic spread operating in a single case is also a possibility as a similar combination of characteristics as in our patient was noted in a case of clear cell carcinoma leading to bladder metastases after 12 years of nephrectomy where the lesion was deep but was located close to the ureteric orifice [15]. These contradictions regarding the mechanism of metastatic spread is nothing unusual in RCC as there are cases of regional recurrence after 31 years [16] and distant metastases 24 years after nephrectomy [17].

First, metastasectomy for RCC was done by Barney in 1939 in a patient with a solitary metastatic lesion in the lung who died 23 years later of cardiac disease [18]. The decision to perform metastasectomy is usually made according to various prognostic criteria: the site and number of metastases, the completeness of resection of the primary tumour, the performance status and the disease-free interval from treatment of the primary tumour to the diagnosis of metastatic disease [19]. Although improved survival after metastasectomy is still debatable, surgical intervention can provide palliation for symptomatic metastatic disease [20].

Endoscopic resection of the bladder metastatic lesion will confirm the diagnosis and may be considered adequate as the primary treatment in cases of drop metastases. Others with deeper lesions would require more extensive surgical treatment including partial or total cystectomy if the metastases are confined to the bladder. Since most of these patients are likely to have systemic metastases, they are given systemic therapy with interleukin-2 or tyrosine kinase inhibitors. The 3-year survival rate of patients with a solitary metastasis is around 80 % [20]. However, about 50 % of patients with multiple metastases have a life expectancy less than 1 year after diagnosis.

## Conclusions

Although rare, papillary renal cell carcinoma can develop metastases in the bladder. Drop metastases, haematogenous spread, embolisation through lymphatics and retrograde venous embolisation are the suggested possible mechanisms. The exact mechanism in a given case appears to be unpredictable. It may be possible that several mechanisms in combination operate in a given case.

## Abbreviations

CT KUB: Computerised tomography of the kidney, ureter and bladder
ECOG: Eastern Cooperative Oncology Group
RCC: Renal cell carcinoma
US scan of KUB: Ultrasound scan of the kidney, ureter and bladder
